# Supplementary material for: Identification and functional analysis of non-coding regulatory small RNA FenSr3 in Bacillus amyloliquefaciens LPB-18
Source: PeerJ. 2023 May 15;11:e15236. doi: 10.7717/peerj.15236 (PMC10194069; doi:10.7717/peerj.15236)
Supplement: Supplemental Information 4 [file peerj-11-15236-s004.zip › KO/CK-vs-T1_map/map05204.html]

KEGG PATHWAY: Chemical carcinogenesis - Reference pathway


|  |  |
| --- | --- |
| **Chemical carcinogenesis - Reference pathway** |  |

[
Pathway menu
| Organism menu
| Pathway entry
| Show description
| User data mapping
]

It has been estimated that exposure to environmental chemical carcinogens may contribute significantly to the causation of a sizable fraction, perhaps a majority, of human cancers. Human carcinogens act through a variety of genotoxic and non-genotoxic mechanisms. Genotoxic carcinogens can attack biological macromolecules such as DNA and RNA either directly or indirectly through metabolism, resulting in the formation of adducts with these macromolecules. If DNA adducts escape cellular repair mechanisms and persist, they may lead to miscoding, resulting in permanent mutations. Non-genotoxic carcinogens act by the mechanisms such as induction of inflammation, immunosuppression, formation of reactive oxygen species, activation of receptors, and epigenetic silencing. Together, these genotoxic and non-genotoxic mechanisms can alter signal-transduction pathways that finally result in hypermutability, genomic instability, loss of proliferation control, and resistance to apoptosis - some of the characteristic features of cancer cells.

|  |  |  |
| --- | --- | --- |
| Reference pathway | 184% 150% 122% 100% 82% 67% 55% | 图片下载 |
